# Sociodemographic disparities and clinical predictors of cochlear implant quality of life: evidence from Saudi Arabia

**DOI:** 10.1038/s41598-026-49062-5

**Published:** 2026-04-20

**Authors:** Faisl M. Alqraini

**Affiliations:** Department of Special Education, Prince Sattam Bin Abdulaziz University, Al-Kharj, Saudi Arabia

**Keywords:** Cochlear implants, Quality of life, Quality of life-35 profile, Saudi Arabia, Socioeconomic status, Health disparities, Health care, Medical research, Psychology, Psychology

## Abstract

Quality of life (QoL) is a critical outcome measure for cochlear implant (CI) users, yet the association between sociodemographic disparities, respondent type, and QoL remains under-explored in the Saudi Arabian context. This study aimed to examine the relationship between sociodemographic and clinical factors specifically respondent type (self-report vs. proxy-report), socioeconomic status, and device usage and CI-related quality of life domains. A cross-sectional observational study was conducted involving 156 participants (CI users and parents/guardians) recruited via digital rehabilitation networks in Saudi Arabia. Data were collected using a structured electronic questionnaire incorporating the modified Cochlear Implant Quality of Life-35 Profile (CIQOL-35). Multiple linear regression models (OLS) were estimated to evaluate factors associated with six QoL domains: communication, emotional, entertainment, environment, listening effort, and social functioning. The regression analysis indicated that family economic status was significantly associated with better outcomes in the communication, environment, and social domains (*p* < .05). Conversely, parents with higher education levels reported significantly lower scores in the communication and environment domains (*β* = − 0.37 and − 0.38, respectively, *p* < .05) compared to those with primary education, potentially reflecting an expectation bias. Notably, respondent type (adult self-report vs. proxy-report) did not demonstrate a statistically significant association with emotional well-being or other domains in this model. Clinical factors such as lack of post-implant habilitation were associated with significantly lower scores across multiple domains. Sociodemographic factors are significantly associated with perceived quality of life among CI users in Saudi Arabia. While families with higher economic resources report better social and environmental outcomes, highly educated parents perceive lower benefits, potentially reflecting differences between clinical outcomes and parental expectations. These findings suggest that rehabilitation programs must integrate psychosocial support that addresses family expectations and socioeconomic barriers to optimize QoL outcomes. Interpretations should be contextualized within the study’s limitations, notably a proxy-dominant sample and the use of a modified instrument not originally validated for pediatric proxy reports.

## Introduction

In Saudi Arabia, the demographic landscape of hearing healthcare is evolving, with cochlear implants becoming an increasingly prevalent intervention for adults and children diagnosed with severe-to-profound hearing loss. These surgically implanted electronic devices are pivotal in providing access to sound as they effectively bypass damaged portions of the ear to stimulate the auditory nerve directly^1^. Recent data indicates a significant expansion of cochlear implantation services across major medical centers in the Kingdom, and this development has substantially increased the accessibility of this life-changing technology for the hearing-impaired population^2^.

However, the efficacy of cochlear implantation extends beyond traditional audiological metrics such as speech perception or sound detection. In this population, Quality of Life has emerged as the central outcome measure for assessing therapeutic success. While the primary function of the device is to improve communication, the broader objective is to enhance emotional well-being, facilitate social functioning, and ensure long-term participation in society^3,4^. Recent local research by Alqraini^5^ has further emphasized that for pediatric populations, these outcomes are critical for holistic developmental and psychosocial adjustment which establishes a vital baseline for understanding cochlear implant efficacy in the region.

Current literature suggests that clinical success does not occur in isolation but is deeply intertwined with the sociodemographic background of the recipient. It is observed that factors such as age, gender, and economic status are inextricably linked with differences in social, emotional, and environmental outcomes^3,4^. This implies that these non-clinical determinants can significantly shape the perceived quality of life for cochlear implant users across various functional domains including communication, entertainment, and listening effort^4^. Despite the growing prevalence of cochlear implants in the region, there remains a need to systematically examine how specific variables, particularly family economic status and the distinction between self-reported versus proxy-reported outcomes, interact within the Saudi context.

## Research questions and goal of the study

Consequently, the primary objective of this study is to examine the complex relationship between these sociodemographic and clinical factors and Cochlear Implant-Related Quality of Life (CIQOL). By characterizing the study population and assessing these associations, this research seeks to delineate the variables associated with functional outcomes. To guide this investigation, the following research questions are proposed:

1. What is the association between sociodemographic factors, including respondent type, gender, age, number of children with cochlear implant, economic status, and parents’ education, and the six domains of CIQOL?
2. What is the link between cochlear implant–related factors, including device use, device provider, child post implant, and satisfaction with services, and the six domains of CIQOL?

## Methodology

### Research design

This study employed a quantitative cross-sectional survey design to examine the factors associated with cochlear implant-related quality of life. This observational approach facilitated the assessment of associations between sociodemographic and clinical variables and quality of life outcomes in a naturalistic setting, capturing the reported experiences of participants without experimental intervention^6^.

### Participants and sampling strategy

A convenience sampling strategy, defined as a non-probability sampling method, was utilized to recruit participants^7^. Recruitment was conducted via digital rehabilitation and support communities on WhatsApp and Telegram, platforms widely used by Saudi families for discussing cochlear implant maintenance and habilitation. The survey was distributed to over 500 members, yielding 156 valid responses. While convenience sampling offers benefits regarding accessibility and practicality in online data collection, it is acknowledged that this strategy may be exposed to response and coverage biases^8^.

The demographic profile indicates that 52.6% of implant recipients were female, with half of the sample (50%) aged between 5 and 13 years. A significant majority (60.3%) underwent implantation at or before the age of 3 years. Socioeconomically, 80.8% of respondents classified their family status as “medium,” and 62% of parents held a university or postgraduate degree. Regarding clinical history, 90.4% received their devices from government providers, and 50% utilized devices from the manufacturer “Cochlear.” (Table 1)

**Table 1:** Sociodemographic and cochlear implant-related factors.

Variable	Category	n	%
Age at implantation	≤ 3 years	94	60.3
	> 3 to ≤ 10 years	55	35.3
	More than 10 years	7	4.5
Does the child receive post-implant habilitation?	No	19	12.2
	Yes	137	87.8
No. of children with CI in family	≤ 2	142	92.8
	3–5	7	4.6
	6 +	4	2.6
	Missing	3	1.9
Current age	≤ 5 years	40	25.6
	> 5 to ≤ 13 years	78	50.0
	> 13 to ≤ 21 years	31	19.9
	More than 21 years	7	4.5
Device provider	Government	141	90.4
	Private/Other	15	9.6
Cochlear device manufacturer	Advanced Bionics	10	6.4
	Cochlear	78	50.0
	MED-EL	62	39.7
	Other	1	0.6
	Unknown	5	3.2
Daily device use (approx. hours)	≤ 5	9	5.8
	6–8	7	4.5
	9–11	30	19.2
	12–14	54	34.6
	15–17	30	19.2
	18 +	26	16.7
Family economic status	Excellent	12	7.7
	Medium	126	80.8
	Low	18	11.5
Gender	Female	82	52.6
	Male	74	47.4
Type of habilitation services	Auditory only	56	35.9
	Other/None	23	14.7
	Speech only	77	49.4
Implant Center	King Abdulaziz University Hospital	49	31.4
	King Fahad Hospital	9	5.8
	King Fahad Medical City	14	9.0
	King Saud Medical City	18	11.5
	Military or National Guard Hospitals	22	14.1
	Others	44	28.2
Parents education	Higher education—University/Postgraduate	97	62.2
	Primary/No formal education	18	11.5
	Secondary/Intermediate	41	26.3
Respondent type	Adult	12	7.7
	Parent or Guardian	144	92.3
Family satisfaction with services	Not Satisfied	9	5.8
	Neutral	26	16.9
	Satisfied	60	39.0
	Very Satisfied	59	38.3
	Missing	2	1.3

#### Sample size determination

An a priori power analysis was conducted using G*Power software (version 3.1.9.7) to determine the requisite sample size. The calculation was based on a linear multiple regression model with 14 predictors (including sociodemographic and clinical variables), an alpha level (*α*) of 0.05, a statistical power of 0.95, and a medium-to-large effect size (*f*^2^ = 0.15). Considering these inputs, the analysis indicated that a minimum sample of 149 participants was necessary to detect significant effects^9^. The final sample of 156 participants exceeded this requirement, ensuring adequate statistical power.

#### Survey instrument

Data were collected using a structured electronic questionnaire divided into two sections. The first section gathered sociodemographic and clinical data, including respondent type, age, and habilitation history. The second section utilized the Cochlear Implant CIQOL-35, a patient-reported outcome measure assessing functional ability across six domains: communication, emotional, entertainment, environmental, listening effort, and social functioning^4^. Items were rated on a 5-point Likert scale ranging from “Never” to “Always.” It is important to note that the CIQOL-35 was not originally validated for proxy or pediatric use, and a specifically validated pediatric or proxy-adapted version was not utilized in this study. Furthermore, subsequent modifications to the instrument, including the removal of specific items to achieve internal consistency, may affect its construct validity and limit direct comparability with existing literature. Rather, the standard instrument was administered to parents and guardians, who were instructed to evaluate the items based on their daily observations of the child’s functional abilities and behaviors across the six domains (Table 2).

**Table 2:** Survey instrument description.

Section	Variables	Number of items	Measurement
Section 1: Socio-demographics and Clinical factors	Respondent type, Gender, Age, Age at Implant, Parents’ Education, Socioeconomic Status, Daily Device Use, Children with CI in Family, Device Provider, Post-Implant Services, Family Satisfaction with Services, Device Manufacturer, Implant Center	Single-item variables	Multiple choice questions
Section 2: CIQOL-35	Communication	10 Items	5-Point Likert Scale (1 = Never, 2 = Rarely, 3 = Sometimes, 4 =
	Emotional	5 Items	
	Entertainment	5 Items	
	Environment	5 Items	
	Listening Effort	5 Items	Often, 5 = Always)
	Social	5 Items	

### Psychometric validation

To ensure measurement integrity, a rigorous psychometric evaluation was conducted using Python. Negatively worded items were reverse-coded prior to analysis. Initial reliability testing using Cronbach’s alpha revealed inconsistencies in specific items, with some values falling below the generally acceptable threshold of 0.70^10^. To address this, the scale was refined by excluding items with poor psychometric properties (inconsistent loading patterns and negative internal consistency).

The revised scale demonstrated robust internal consistency, with Cronbach’s alpha coefficients exceeding 0.70 for all constructs. Furthermore, all constructs achieved Composite Reliability (CR) scores greater than 0.70 and Average Variance Extracted (AVE) values above 0.50, confirming strong convergent validity for the modified instrument (Table 3).

**Table 3:** Construct reliability and validity.

Construct	No. of items	Cronbach’s α	Composite reliability (CR)	Average variance extracted (AVE)
Communication	10	0.84	0.95	0.65
Emotional	3	0.73	0.85	0.66
Entertainment	4	0.88	0.92	0.74
Environment	5	0.89	0.93	0.72
Listening Effort	3	0.77	0.87	0.70
Social	4	0.86	0.91	0.71

All Cronbach’s and CR values exceed 0.70, indicating high internal consistency. All AVE values exceed 0.50, indicating strong convergent validity. Items with inconsistent loadings (EMO1, EMO5, ENT1, LE4, LE5, SOC2) were excluded to optimize the measurement model.

### Data analysis

Statistical analyses were performed using Python. Descriptive statistics were computed to summarize participant characteristics. To address the research questions, multiple linear regression models (Ordinary Least Squares) were estimated for each of the six CIQOL domains. Regression model assumptions, including linearity, residual behavior, and homoscedasticity, were assessed and deemed acceptable prior to final model estimation. The models incorporated continuous predictors (e.g., age at implantation, device use hours) and categorical predictors (e.g., respondent type, device provider), with the latter dummy-coded against the most frequent category. Heteroskedasticity-robust standard errors (HC3) were employed to ensure the stability of the estimates.

Predictor variables were selected a priori based on clinical relevance and established theoretical frameworks, rather than automated model reduction strategies, to fully evaluate the targeted sociodemographic and clinical factors. With a sample size of 156 and 14 independent predictors, the observation-to-predictor ratio was approximately 11:1. This exceeds the conventional minimum threshold of 10 observations per variable, thereby providing adequate statistical stability and appropriately mitigating the risk of model overfitting.

## Results

Regression Analysis of CIQOL Domains Multiple linear regression analyses were conducted to identify the significant predictors of quality of life across the six CIQOL domains. The models evaluated the influence of sociodemographic factors and clinical variables on participant outcomes. The detailed regression coefficients are presented in Table 4.

**Table 4:** Multiple linear regression coefficients across CIQOL domains.

Predictors	Com	Emo	Ent	Env	List	Soc
Intercept	2.64***	3.12***	3.07***	3.09***	2.72***	4.11***
Respondent Type *(Ref: Parent)*						
Adult	− 0.04	0.07	− 0.53	− 0.07	0.27	− 0.50
Age at Implantation	0.01	− 0.06*	0.04	0.03	0.05	− 0.02
Device Use (Daily)	0.03*	0.02	0.04	0.05*	0.05*	− 0.01
No. of Children with CI	0.14	0.04	0.13	− 0.02	− 0.11	− 0.13
Gender *(Ref: Female)*						
Male	− 0.08	0.07	− 0.07	0.17	0.08	0.27
Device Provider *(Ref: Govt)*						
Private/Other	0.23	− 0.27	0.02	0.24	0.30	− 0.08
Economic Status *(Ref: Medium)*						
Excellent	0.48**	0.53	0.31	0.37*	0.40	0.69**
Low	− 0.23	− 0.16	− 0.51	− 0.34	− 0.37	− 0.25
Child Post-Implant *(Ref: Yes)*						
No	− 0.49**	− 0.52	− 0.65*	− 0.75**	− 0.53*	− 0.54
Satisfaction *(Ref: Satisfied)*						
Neutral	− 0.14	− 0.06	− 0.19	− 0.07	− 0.24	0.10
Not Satisfied	− 0.38	− 0.60	− 0.07	0.05	− 0.15	− 0.31
Very Satisfied	0.30*	0.25	0.43*	0.36*	0.22	0.30
Parents’ Education *(Ref: University)*						
Primary/No formal	0.51**	− 0.33	0.10	0.47*	0.39	0.09
Secondary/Intermediate	− 0.24	− 0.01	− 0.35	− 0.31	− 0.42*	0.23
N	151	151	151	151	151	151
R^2^	0.40	0.19	0.28	0.31	0.28	0.17

Com., Communication; Emo., Emotional; Ent., Entertainment; Env., Environment; List., Listening Effort; Soc., Social.

*p < 0.05, **p < 0.01, ***p < 0.001.

### Sociodemographic factors

In this model, respondent type (Adult self-report vs. Parent proxy) did not emerge as a statistically significant predictor across the six domains (e.g., Emotional domain: *β* = 0.07, *p* > 0.05), suggesting that within this sample, the mode of reporting did not systematically skew the quality of life scores.

### Family economic status

Socioeconomic standing emerged as a robust positive predictor. Participants from families with “Excellent” economic status reported significantly better outcomes in Communication (*β* = 0.48, *p* < 0.01), Environment (*β* = 0.37, *p* < 0.05), and Social Functioning (*β* = 0.69, *p* < 0.01) compared to the reference group. This indicates that financial resources may facilitate better access to supportive environments and social opportunities that enhance the utility of the implant.

### Parental education

Conversely, parental education exhibited an inverse relationship with perceived outcomes. Parents with university-level education (the reference group) tended to report lower scores than those with primary education, specifically in the Communication (*β* = 0.51 for Primary vs. University) and Environment domains. This finding may reflect higher baseline expectations among more educated parents regarding the device’s efficacy, leading to a stricter evaluation of the child’s progress.

### Clinical factors

The absence of post-implant habilitation services was strongly associated with negative outcomes. Participants who did not receive habilitation scored significantly lower in Communication (*β* =  − 0.49, *p* < 0.01), Entertainment (*β* =  − 0.65, *p* < 0.05), and Environment (*β* =  − 0.75, *p* < 0.01), reinforcing the clinical necessity of structured auditory training.

## Discussion

This study investigated the sociodemographic and clinical factors associated with CIQOL in Saudi Arabia. The findings demonstrate that perceived quality of life is significantly associated with non-clinical factors, specifically family economic status and parental expectations.

### Alignment of self-report and proxy-report

Contrary to initial hypotheses derived from some literature, this study did not find a statistically significant difference in quality of life scores between adult users (self-report) and parents (proxy-report). However, given the small sample size of adult respondents (*n* = 12), there was severely limited statistical power to detect meaningful differences between proxy and self-reported outcomes. Therefore, rather than implying strict equivalence or accuracy between self-reports and proxy assessments, the current findings must be interpreted with caution, as they predominantly reflect the parental perception of quality of life rather than a balanced integration of both perspectives.

Furthermore, the heavy reliance on proxy reporting introduces specific challenges regarding the validity of subjective domains. While parents and guardians are well-positioned to observe external, functional abilities, such as a child’s communicative responses or environmental awareness, they may be less accurate in assessing internal states, such as emotional well-being and nuanced social functioning. This inherent limitation in proxy assessment of subjective experiences may introduce measurement noise, which is potentially reflected in the statistical modeling; notably, the regression model for the Emotional domain yielded a comparatively lower coefficient of determination (*R*^2^ = 0.19). Future research must acknowledge that while proxy reports provide vital caregiver perspectives, they remain an indirect measure of the psychosocial and emotional realities experienced by the cochlear implant user.

### Socioeconomic status and resource accessibility

Family economic status emerged as a robust positive predictor for the Communication, Environment, and Social domains. This association is consistent with global findings indicating that families with higher socioeconomic resources are better positioned to provide a rich auditory environment and access supplementary support^4^. In the Saudi context, where government-funded healthcare is widely available, this finding suggests that financial stability likely correlates with the ability to maximize the device’s utility through extracurricular social engagement and reduced environmental stressors^3^.

Beyond providing a richer auditory environment, this socioeconomic advantage fundamentally alters the continuity of care. In the Saudi context, while the device and initial surgeries are frequently covered by government healthcare, the logistical demands of long-term habilitation such as transportation to specialized urban centers, time taken off work for frequent appointments, and the ability to finance private, supplementary speech-language therapy when public waitlists are long remain heavily dependent on family resources. Therefore, the higher CIQOL scores reported by families with excellent economic status likely reflect their capacity to overcome these systemic barriers and maintain uninterrupted, high-frequency rehabilitation.

### The paradox of parental education

This study found a significant negative association between university-level education and scores in the Communication and Environment domains relative to primary education. This phenomenon can be interpreted through the lens of “expectation bias.” Highly educated parents often possess elevated expectations regarding the technological capabilities of the implant, anticipating near-normal hearing restoration^5^. When the child’s progress, while clinically successful, does not match these high aspirations, parents may rate the functional outcomes more critically than parents with lower formal education. This suggests a clinical imperative to manage parental expectations effectively during the pre-implantation counseling phase.

Furthermore, this inverse relationship extends beyond mere expectation bias into the realm of health literacy. Highly educated parents frequently engage more deeply with complex developmental literature and normative milestones. Consequently, they may be more acutely aware of subtle linguistic or environmental deficits that a less-educated parent might overlook. This heightened sensitivity can result in more rigorous, critical self-reporting on the CIQOL-35. Thus, the lower scores reported by university-educated parents may not solely indicate poorer clinical outcomes, but rather a more stringent evaluation metric applied by caregivers with advanced health literacy.

### Clinical factors and habilitation

Consistent with established protocols, the lack of post-implant habilitation was negatively associated with outcomes across almost all domains. This reinforces the consensus that while the device provides access to sound, consistent rehabilitation is what assigns functional meaning to that sound^1^. Crucially, the necessity of this habilitation spans across age groups, though its specific role shifts. For early-implanted pediatric cohorts, structured auditory-verbal therapy is foundational for initial language acquisition and neuroplastic development. For older, late-implanted children or adults, habilitation remains equally vital, shifting focus toward auditory training, complex listening effort management, and social integration strategies. Across all demographics, the absence of this structured follow-up severely compromises the overarching quality of life.

## Limitations

Several limitations should be considered. First, the study employed a cross-sectional design, which limits causal inferences. Second, the reliance on convenience sampling via digital platforms may introduce selection bias. Third, the respondent composition was heavily skewed toward parental proxy reports, with a very small number of adult self-reports (*n* = 12). While this reflects the engagement demographics of the recruited digital networks, it limits the statistical power to detect specific effects within the adult subgroup and inherently biases the overall findings toward caregiver perspectives. Consequently, subjective domains particularly emotional and social functioning may reflect parental observation and expectation rather than the direct internal experiences of the users, limiting the generalizability of these results to the adult cochlear implant population. Fourth, the CIQOL-35 was not originally validated for proxy or pediatric use, its utilization for proxy reporting carries implications for measurement validity. Additionally, modifications made to the instrument specifically the removal of several items to meet reliability thresholds may affect construct validity and limit the ability to directly compare these findings with existing literature that employs the standard CIQOL-35. While parents can accurately observe external behaviors related to communication and environment, applying adult-centric items to pediatric populations via proxy may introduce measurement variance.

## Conclusion

Sociodemographic factors are significantly associated with perceived quality of life among cochlear implant users in Saudi Arabia. While families with higher economic resources report better social and environmental outcomes, highly educated parents perceive lower benefits, likely due to higher baseline expectations. These findings suggest that clinical success cannot be measured solely by audiometric thresholds. Rehabilitation programs may benefit from being tailored to address the distinct psychosocial needs of families and should explicitly address parental expectations particularly among highly educated families to align their anticipations with realistic prognostic outcomes.

## Data Availability

The datasets generated and analyzed during the current study are not publicly available to protect participant confidentiality but are available from the corresponding author upon reasonable request.
